# Comparative Study of Negative Capacitance Field-Effect Transistors with Different MOS Capacitances

**DOI:** 10.1186/s11671-019-3013-z

**Published:** 2019-05-24

**Authors:** Jing Li, Yan Liu, Genquan Han, Jiuren Zhou, Yue Hao

**Affiliations:** State Key Discipline Laboratory of Wide Band Gap Semiconductor Technology, School of Microelectronics, Xidian University, Xi’an, 710071 People’s Republic of China

**Keywords:** Germanium, Negative capacitance, Passivation time

## Abstract

We demonstrate the negative capacitance (NC) effect of HfZrO_x_-based field-effect transistors (FETs) in the experiments. Improved *I*_DS_, SS, and *G*_m_ of NCFET have been achieved in comparison with control metal oxide semiconductor (MOS) FET. In this experiment, the bottom MIS transistors with different passivation time are equivalent to the NC devices with different MOS capacitances. Meanwhile, the electrical properties of NCFET with 40 min passivation are superior to that of NCFET with 60 min passivation owing to the good matching between *C*_FE_ and *C*_MOS_. Although SS of sub-60 mV/decade is not achieved, the non-hysteretic transfer characteristics beneficial to the logic applications are obtained.

## Introduction

With the scaling down of transistor, the integration level of integrated circuit (IC) is continuous growing. An accompanying power dissipation problem is urgent to be solved. In order to circumvent this problem, the operation voltage of the transistor should be reduced [1]. The subthreshold swing (SS) of MOSFET cannot be below 60 mV/decade at room temperature, which restricts the reduction of threshold voltage *V*_TH_ and supply voltage *V*_DD_ [2]. Many efforts have been devoted to the research and the development of devices with novel transport and switching mechanisms to beat the Boltzmann limit, including negative capacitance field-effect transistor (NCEFT) [3, 4], resistive gate FET [5], nano-electro mechanical FET (NEMFET) [6, 7], impact ionization metal-oxide-semiconductor (I-MOS) [8, 9], and tunneling FET [10, 11]. Among them, NCFET has aroused much attention because it can achieve a steep SS without losing the drive current [12–15]. Doped HfO_2_ (e.g., HfZrO_*x*_ (HZO) and HfSiO_*x*_) has been widely used in NCFETs [4, 16, 17]; it is compatible with the CMOS process [18]. A theoretical study has shown that the undesired hysteresis occurs due to unmatched ferroelectric capacitance *C*_FE_ to underlying MOS capacitance *C*_MOS_ in NCFET [19]. However, the effect of matching between *C*_FE_ and *C*_MOS_ on the electrical characteristics of NCFETs is still a concern in the experiments.

In this work, the electrical characteristics of NC Ge FETs with different MOS capacitances are studied based on the different matching between *C*_FE_ and *C*_MOS_. Although SS less than 60 mV/decade does not appear, the hysteresis-free transfer characteristics and better electrical properties are obtained. Apparent peaks of *C*_FE_ versus *V*_FE_ curves demonstrate NC effect of HZO based NCFETs. The better matching of *C*_FE_ and *C*_MOS_ contributes to steeper SS and higher on current, which is beneficial to the logic applications.

## Methods

**Fig. 1:**
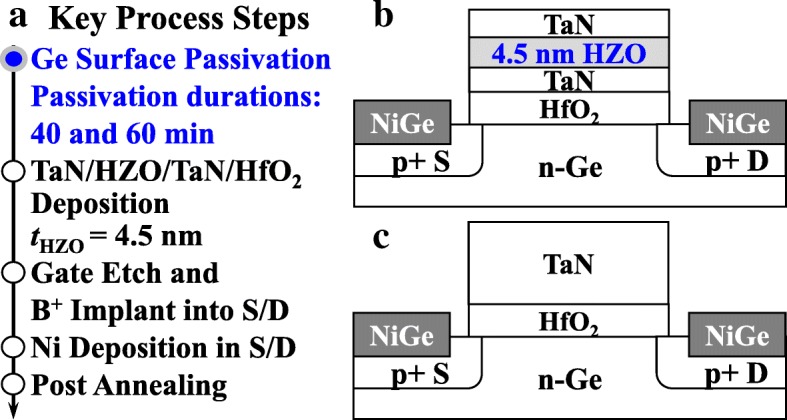
**a** Key process steps of fabricated NC devices. The schematics of the fabricated **b** NCFET and **c** control MOSFET

The key fabrication process of Ge NCFETs is shown in Fig. 1a. Four-inch n-Ge(001) wafers with a resistivity of 0.088–0.14 Ω·cm were used as the starting substrates. After pre-gate cleaning, Ge wafers were loaded into an ultra-high vacuum chamber for surface passivation using Si_2_H_6_. Two passivation durations of 40 and 60 min were used. Then, TaN/HZO/TaN/HfO_2_ stack was deposited. The thicknesses of the HfO_2_ dielectric layer and HZO FE layer are 4.35 and 4.5 nm, respectively. After gate patterning and etching, source/drain (S/D) regions were implanted using boron ions (B^+^) at an energy of 30 keV and a dose of 1 × 10^15^ cm^−2^. S/D metal Nickel was formed using a lift-off process. Finally, rapid thermal annealing at 450 °C for 30 s was carried out. Control MOSFET with TaN/HfO_2_ stack was also fabricated. Figures 1b and c show the schematics of fabricated NCFET and control MOSFET, respectively. The internal metal gate in the fabricated NCFET counterbalances the potential at the channel surface, which is called the MFMIS structure.

## Results and Discussion

**Fig. 2:**
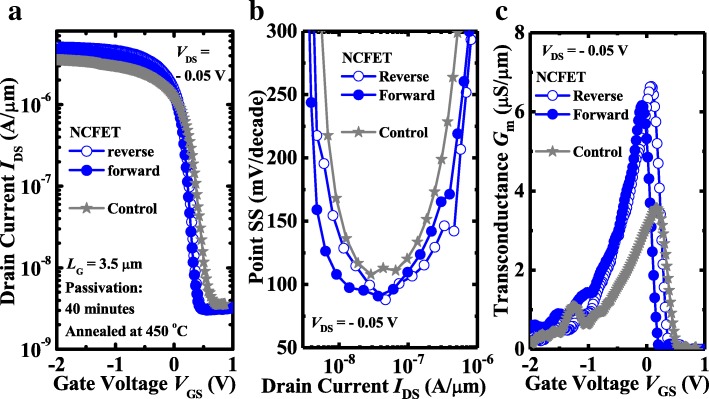
**a** The measured *I*_DS_-*V*_GS_ curves of the NCFET and control MOSFET with 40 min passivation. Comparison of **b** point SS versus *I*_DS_ and **c** *G*_m_ characteristics between NC FET and control MOSFET

**Fig. 3:**
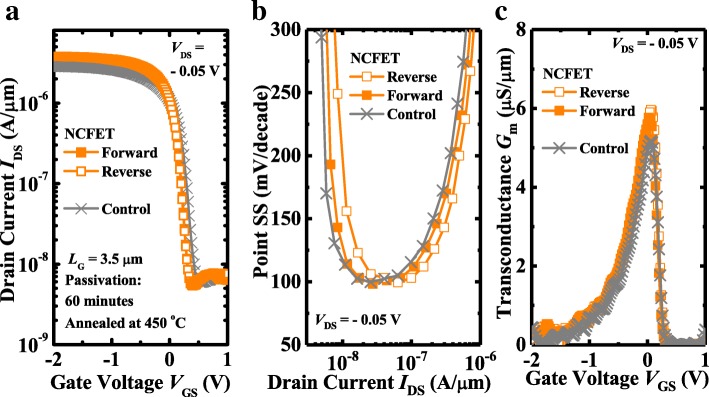
**a** The measured *I*_DS_-*V*_GS_ curves of the NCFET and control MOSFET with 60 min passivation. Comparison of **b** point SS versus *I*_DS_ and **c** *G*_m_ characteristics between NCFET and control MOSFET

Figure 2a plots the measured *I*_DS_-*V*_GS_ curves of a pair of NCFET and control MOSFET with 40 min surface passivation. Both devices have a gate length *L*_G_ of 3.5 μm. The NC device with 40 min passivation has a significantly improved *I*_DS_ than the control MOSFET. The transfer curves of NCFET exhibit a non-hysteretic feature. Point SS versus *I*_DS_ curves in Fig. 2b show that the NC transistor has improved SS over the control device, although SS of sub-60 mV/decade does not appear. Figure 2c shows that NC transistor obtains a significantly boosted linear transconductance *G*_m_ over the control device at *V*_DS_ of − 0.05 V. Figure 3 compares the electrical performances of NCFET and control MOSFET with surface passivation for 60 min. Similarly, the *I*_DS_, point SS and *G*_m_ of NCFET are superior to that of control MOSFET.

**Fig. 4:**
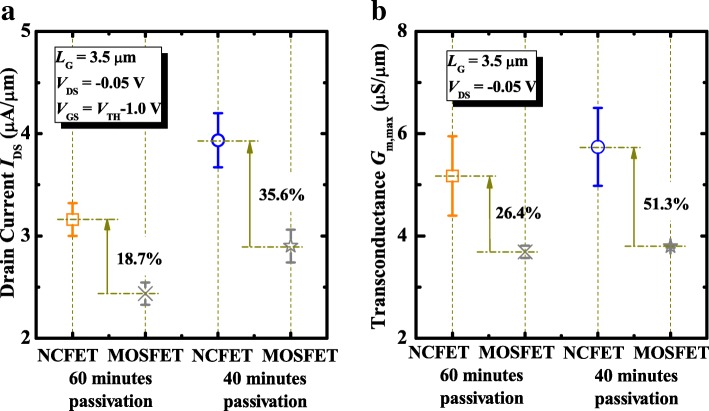
The statistical **a** *I*_DS_ and **b** *G*_m_ results of NCFETs and control MOSFETs with 40 and 60 min passivation durations

Figure 4a shows the statistical results of the drive current of NCFETs and control MOSFETs at *V*_DS_ of − 0.05 V and *V*_GS_-*V*_TH_ = − 1.0 V. NCFETs demonstrate 18.7% and 35.6% improvement in *I*_DS_ for the 60 min and 40 min surface passivation, respectively, in comparison with the control devices. It is speculated that the NCFETs passivated for 40 min have a better matching between *C*_MOS_ and *C*_FE_ over the NC devices with 60 min. Figure 4b shows that NCFETs obtain 26.4% and 51.3% improvement in maximum transconductance *G*_m,max_ for 60 min and 40 min surface passivation, respectively, in comparison with the control devices. It is seen that the control MOSFETs with surface passivation for 40 min have a higher *I*_DS_ and *G*_m,max_ than the devices passivated for 60 min, which is due to the larger *C*_MOS_ induced by the smaller equivalent oxide thickness (*E*_OT_). The internal metal gate provides an equipotential plane; the device can be equivalently modeled as a capacitive voltage divider. The total capacitance *C*_G_ is a series of *C*_FE_ and *C*_MOS_. The internal gate voltage is amplified owing to the NC effect. The internal voltage amplification coefficient *β* =  ∣ *C*_*FE*_ ∣ / ∣ *C*_*FE*_ ∣  − *C*_*MOS*_ gets the maximum when |*C*_MOS_| = |*C*_FE_| [20, 21]. Achieving the optimized matching of *C*_FE_ and *C*_MOS_ is the prerequisite of the improvement of on current.

**Fig. 5:**
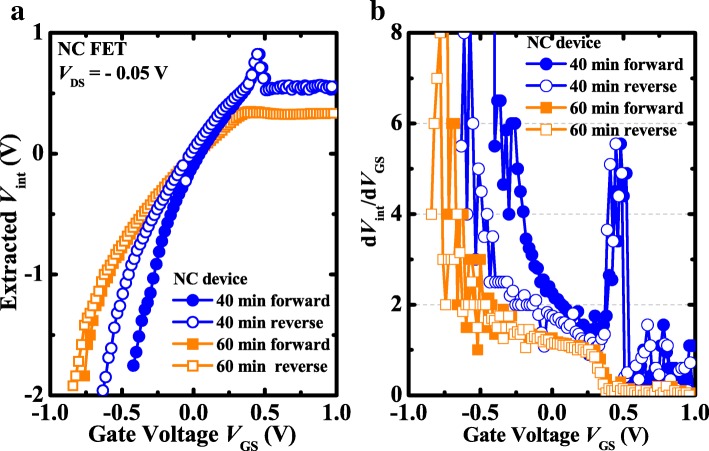
**a** Extracted *V*_int_ as a function of *V*_GS_ curves. **b** The internal voltage amplification coefficient versus *V*_GS_ curves

The extracted *V*_int_ versus gate voltage *V*_GS_ curves are shown in Fig. 5a. *V*_int_ of NC transistor can be extracted on account of the hypothesis that *I*_DS_-*V*_int_ curve of NC transistor is exactly identical with *I*_DS_-*V*_GS_ curve of the control device. The internal voltage amplification coefficient *dV*_int_/*dV*_GS_ is shown in Fig. 5b. d*V*_int_/d*V*_GS_ > 1 is achieved in the wide sweeping range of *V*_GS_ for the NCFET with 40 min surface passivation, contributing to a steeper SS than the control device during the measuring process, which is due to the local polarization switching [22]. It is consistent with the aforementioned results in Fig. 2b. For the NCFET with 60 min passivation, the internal voltage amplification coefficient d*V*_int_/d*V*_GS_ > 1 is achieved during the range of *V*_GS_ < 0 V for the double sweeping of *V*_GS_, which is in agreement with the elevated SS in Fig. 3b.

**Fig. 6:**
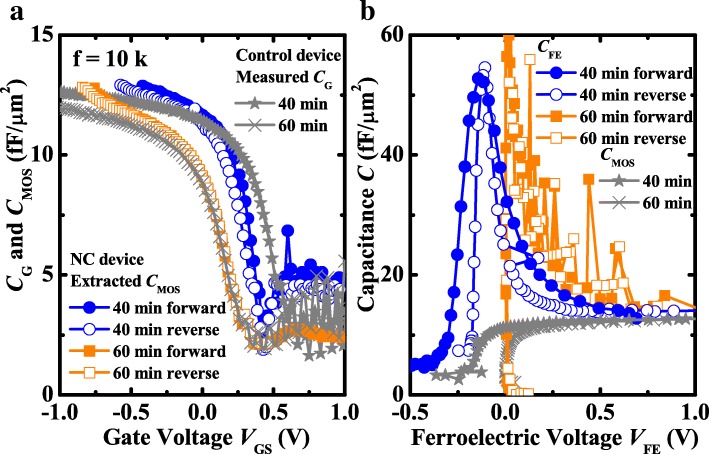
**a** Measured *C*_G_ and extracted *C*_MOS_ as a function of *V*_GS_. **b** *C*_FE_ and *C*_MOS_ versus *V*_FE_ curves

Figure 6a shows the extracted *C*_MOS_ versus *V*_GS_ curves for NC transistor, which is relying on the *V*_int_-*V*_GS_ in Fig. 5a and the *C*_G_-*V*_GS_ curves of control MOSFETs. The extracted *C*_MOS_ is in good agreement with the measured *C*_G._ Hence, the validity of the calculation method is demonstrated. The *C*_FE_ and *C*_MOS_ versus *V*_FE_ curves are depicted in Fig. 6b. From the initiation of NC effect, the absolute value of negative *C*_FE_ of the transistor exceeds *C*_MOS_ for double sweeping of *V*_GS_ all the time in Fig. 6b. |*C*_FE_| > *C*_MOS_ and *C*_FE_ < 0 can cause hysteresis-free characteristics, and the matching of *C*_MOS_ and *C*_FE_ is beneficial to the logic applications [23, 24]. Hysteresis-free characteristics in Figs. 2a and 3a are observed attributed to all the domain matching and inhibited charge trapping [25]. The stable polarization switching is responsible for the non-hysteretic characteristics [26]. Furthermore, the large internal gate gain d*V*_int_/d*V*_G_ > 1 is ascribed to the slight discrepancy between |*C*_FE_| and *C*_MOS_ in the subthreshold region, resulting in the steep SS of NC device. Meanwhile, there is a better matching between *C*_FE_ and *C*_MOS_ for the NCFET with 40 min passivation than the NCFET with 60 min passivation. Thus, this provides direct evidence to indicate that the NCFET with 40 min passivation possesses a better electrical performance than the NCFET with 60 min passivation. The FE polarization changes the *V*_FE_; hence the charge of FE varies. The total charge multiplies, which is attributed to the FE polarization besides the increment of *V*_GS_. In other words, for the given *V*_GS_, the charge in the channel increases so the *I*_DS_ improves. As a consequence, the steep SS of transfer characteristic appears in the experiments.

## Conclusions

The hysteresis-free transfer characteristics are obtained for the NCFETs with 40 and 60 min passivation. NC Ge pFETs with 40 min passivation have better electrical characteristics than the NC device with 60 min passivation in experiments. We also demonstrate the NC effect of HZO based NCFETs. For NCFETs, the steep SS and d*V*_int_/d*V*_GS_ > 1 are obtained. The NCFET with 40 min passivation has achieved a good matching between *C*_FE_ and *C*_MOS_, which contributes to the non-hysteretic characteristics. The different NC behaviors are considered to be related to the microscopic domain wall switching in the FE thin films.

## Data Availability

The datasets supporting the conclusions of this article are included in the article.

## Abbreviations

B^+^: Boron ions
*E*_OT_: Equivalent oxide thickness
FETs: Field-effect transistors
HZO: HfZrO_*x*_
IC: Integrated circuit
I-MOS: Impact ionization metal-oxide-semiconductor
MOS: Metal oxide semiconductor
NC: Negative capacitance
NCFET: Negative capacitance field-effect transistor
NEMFET: Nano-electro mechanical FET
S/D: Source/drain
SS: Subthreshold swing
